# The effectiveness of a life style modification and peer support home blood pressure monitoring in control of hypertension: protocol for a cluster randomized controlled trial

**DOI:** 10.1186/1471-2458-14-S3-S4

**Published:** 2014-11-24

**Authors:** Tin Tin Su, Hazreen Abdul Majid, Azmi Mohamed Nahar, Nurul Ain Azizan, Farizah Mohd Hairi, Nithiah Thangiah, Maznah Dahlui, Awang Bulgiba, Liam J Murray

**Affiliations:** 1 Centre for Population Health (CePH), Department of Social and Preventive Medicine, Faculty of Medicine, University of Malaya, Kuala Lumpur, Malaysia; 2 Department of Sports Medicine, Faculty of Medicine, University of Malaya, Kuala Lumpur, Malaysia; 3 Julius Centre University of Malaya (JCUM), Department of Social and Preventive Medicine, Faculty of Medicine, University of Malaya, Kuala Lumpur, Malaysia; 4 Centre for Public Health, Queen's University of Belfast, Belfast, Ireland

**Keywords:** Lifestyle modification, self-blood pressure monitoring, hypertension, cluster randomized controlled trial, Malaysia

## Abstract

**Background:**

Death rates due to hypertension in low and middle income countries are higher compared to high income countries. The present study is designed to combine life style modification and home blood pressure monitoring for control of hypertension in the context of low and middle income countries.

**Methods:**

The study is a two armed, parallel group, un-blinded, cluster randomized controlled trial undertaken within lower income areas in Kuala Lumpur. Two housing complexes will be assigned to the intervention group and the other two housing complexes will be allocated in the control group. Based on power analysis, 320 participants will be recruited. The participants in the intervention group (n = 160) will undergo three main components in the intervention which are the peer support for home blood pressure monitoring, face to face health coaching on healthy diet and demonstration and training for indoor home based exercise activities while the control group will receive a pamphlet containing information on hypertension. The primary outcomes are systolic and diastolic blood pressure. Secondary outcome measures include practice of self-blood pressure monitoring, dietary intake, level of physical activity and physical fitness.

**Discussion:**

The present study will evaluate the effect of lifestyle modification and peer support home blood pressure monitoring on blood pressure control, during a 6 month intervention period. Moreover, the study aims to assess whether these effects can be sustainable more than six months after the intervention has ended.

## Background

Data reported from the World Health Organization (WHO) showed that hypertension caused approximately 13% of annual deaths worldwide in the year of 2004. Death rates due to hypertension in low and middle income countries are higher compared to high income countries [1,2]. Based on a report by WHO's South East Asia, hypertension prevalence ranges from 8% to 40% in the region. Hypertension is also a major non communicable disease in Malaysia. It is estimated that 5.8 million people or 21% of the entire Malaysian population suffer from hypertension and there was an increasing trend over the past decade [3].

Despite availability of effective anti-hypertensive medications at affordable cost, the control of hypertension is still a major issue in both developed and developing countries in Asia [4]. Lifestyle modification which mainly includes dietary adjustment, exercise and weight management can be used as a non-pharmacological intervention in managing chronic non communicable diseases [5]. According to previous evidence, life style modification is a promising tool for prevention and control of hypertension [6-9].

Patients' non-cooperation or lack of compliance to pharmacological and non-pharmacological treatment are identified as key barriers in hypertension management [10]. A review by Glynn et al (2010) concluded that self-monitoring of blood pressure and regular follow up increased adherence to medication as well as blood pressure control. It was supported by several studies that self-management of hypertensive patients which includes home blood pressure monitoring aids in blood pressure reduction [11-13].

The present study is designed to combine life style modification and home blood pressure monitoring for control of hypertension in the context of low and middle income countries. To our knowledge, this is the first study to explore the effect of peer support home blood pressure monitoring rather than an individual based approach. The outcome from this study potentially may assist in development of future public health intervention in a resource limited setting.

## Materials/design

### Objectives

Objectives of this study are to investigate the effect of lifestyle modification and peer support home blood pressure monitoring on blood pressure control, dietary intake, physical activity and fitness during asix-month intervention period. Moreover, we aim to assess whether these effects are sustained more than six months after the intervention has ended.

### Study design and setting

**Figure 1:**
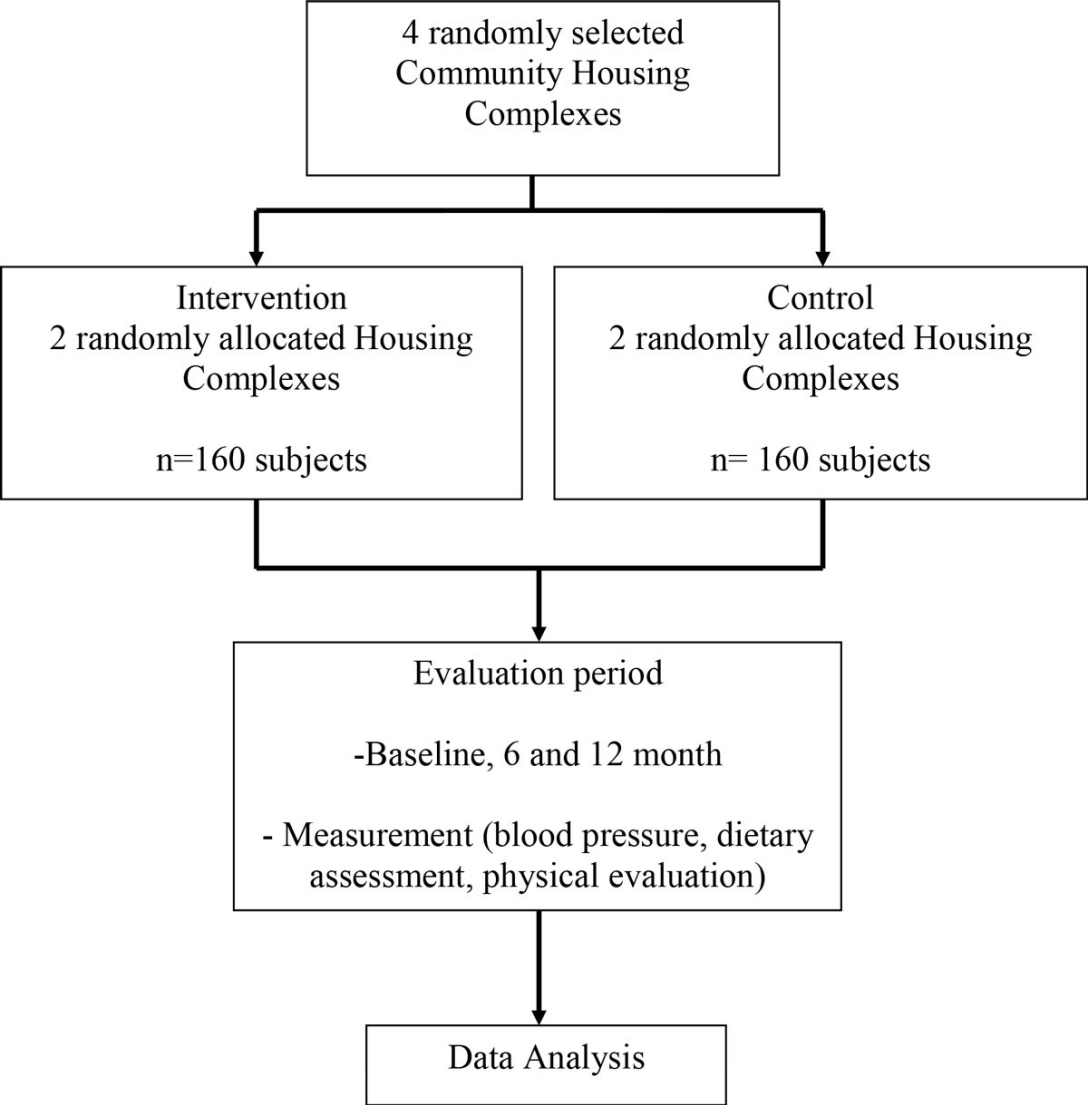
Flow of participants in the intervention and control arm

The study is a two armed, parallel group, un-blinded, cluster randomized controlled trial undertaken within lower income areas in Kuala Lumpur. There are six community housing complexes in the study area. Of these four housing complexes will be randomly selected and allocated (1:1) to the intervention and control arm (Figure 1).

The intervention group will receive training on self-measurement of blood pressure, health coaching on healthy diet, training for indoor exercise activities and the use of Body Mass Index (BMI) calculator. Participants in the control group will receive a BMI calculator and a pamphlet developed by the study team which includes brief information on hypertension, healthy diet and physical activity.

Residents of the selected community housings who were diagnosed as having (stage 1 to stage 3) hypertension by medical professionals will be recruited as participants for the study. The potential participants will be identified based on the household survey data which was carried out by the 'PARTNER' study team from the University of Malaya. This survey was a cross-sectional design and conducted between February to November 2012. Altogether, 833 households were recruited from total 4726 households of four Community Housing Projects by using simple random sampling method. From 833 households, 431 individuals with hypertension were identified.

Inclusion and exclusion criteria of the study are as follows.

#### Inclusion criteria

Adults aged 18 years and above will be eligible for the study if they have been diagnosed as having hypertension (stage 1 to 3) by medical professionals, Malaysian citizen, able to speak Malay (national language) or/and English, willing to participate in all aspects of the intervention and fit to do exercise. Participants who can do daily activities without limitations are defined as "Fit to do exercise". The potential participants will be screened by Physical Activity Readiness Questionnaire (PAR-Q) whether they are fit to do exercise or need further consultation with physician[14].

#### Exclusion criteria

Residents who are pregnant, participants of other clinical trials or plan to leave the area before end of the study period will be excluded.

### Sample size calculation and recruitment

The sample size was calculated by assuming p < 0.05 as the significance level, 80% power and 1 to 1 as the ratio of unexposed to expose in sample. The calculation was carried by using the OpenEpi version 2.3.1 [15]. On the assumption of an SD of 15 mmHg and 30% drop out, a sample size of 160 participants per group is required to detect the effect size of systolic blood pressure 5.4 mm Hg. The effect size used to calculate the sample size was based onthe result of a previous similar study[16].

Universal sampling method will be used to recruit the study participants. The 431 residents of the selected community housing complexes who were diagnosed as (stage 1 to 3) hypertension will be invited to the study. We will take all participants which fit with inclusion criteria and willing to participate to the study so that actual participants may be more than calculated sample size.

### Intervention

The intervention is developed based on Social Cognitive Theory which includes social support, self-regulation and self-efficacy [17]. There will be three main components in the intervention i) Peer support for home blood pressure monitoring, ii) Face to face health coaching on healthy diet iii) Demonstration and training for indoor home based exercise activities.

Peer support groups, consisting of five persons per group will be formed based on the proximity of the residences. An Omron digital blood pressure monitor (HEM-7111) will be distributed to each group and participants will be trained by a state registered nurse on how to measure blood pressure and record the blood pressure reading in a log book.

Dietary assessments of individual participants will be conducted by three dietitians using 7 days diet histories. These dietitians will be randomly assigned to participant groups to assess the food intake. The same principal dietitian will conduct six sessions of health coaching to the participants in the group. The content of health coaching will include diet modification according to the DASH (Dietary Approaches to Stop Hypertension)diet [7,18] and reduction of sodium intake. The sodium reduction approach that will be used in this study is by educating the participants to use herbs and spices in their cooking and by reducing the table salt usage. Healthy cooking demonstration and practices of reading food label will also be included in the sessions of health coaching. High in sodium foods will be encouraged to be minimised. The health coaching materials to the intervention groups are the same whilst control will receive healthy diet leaflet at the beginning of the study. After completing the intervention, the three dietitians will do the post intervention dietary assessment using the 7 days diet histories.

Exercise demonstrations and training will be conducted by a certified trainer from the sport medicine department. The exercise demonstration will focus on strengthening and flexibility exercise. The participants will be recommended to do low to moderate level physical activity and exercise minimum 150 minutes per week. Brisk walking will be also recommended as aerobic exercise. An exercise compact disc (CD) will be provided to the study participants as a guide to do exercise on their own. The participants will also be trained to record their physical activity in a log book. A Body Mass Index (BMI) calculator will be provided to help them to check their own BMI.

### Study tools and measurements

The following tools and measurements will be used to evaluate the effect of the intervention and compliance of the participants regarding lifestyle modification included in the intervention. The measurements will be taken at baseline, six months and twelve months for both groups.

#### Blood pressure measurement

Systolic and diastolic arterial blood pressure will be measured by using Omron Automated Blood Pressure Monitor HEM-7211 which is recommended by the Malaysian Hypertension Association. The arterial blood pressure measurement will be done according to the standardized procedure recommended by World Health Organization [19]. After a rest of ten minutes, two sitting blood pressure will be taken 5 minutes apart on either arm. The average of these two readings will be used as the BP reading of the individual.

#### Questionnaire booklet

A booklet of self-administered questionnaires will be provided for the participants to be completed. Questionnaires include i) Socio-demographic characteristics, ii) Knowledge, awareness and treatment of hypertension, and iii) Global Physical Activity Questionnaire (GPAQ) and, iv) Socio Cognitive variables.

The questionnaire for knowledge, awareness and treatment of hypertension is adapted from the questionnaire developed by World Health Organization [20]. The questionnaire has been translated into Malay version. Face validity has been conducted with 30 participants. The results from the pilot testing showed that contents of the questionnaire are understandable to the participants and can be applied for targeted population.

GPAQ is a tool developed by World Health Organization. It consists of 16 questions to measure the level of physical activity of each individual. The total score of physical activity will be expressed as METs (Metabolic Equivalents). One MET is defined as the energy cost of sitting quietly, and is equivalent to a caloric consumption of 1 kcal/kg/hour. In order to calculate a person's overall energy expenditure, 4 METs will get assigned to the time spent in moderate activities, and 8 METs to the time spent in vigorous activities [21]. The GPAQ questionnaire has been translated and validated into Malay version [22]

#### Dietary assessment

7 Days Diet History is chosen for dietary assessment. The tool has been pre-tested with 20 subjects from the community housing complexes, 10 with hypertension and 10 without hypertension. The 7 Days Diet History will be conducted by trained dietitians. A diet history flip chart will be used as a supplementary tool to assist the study participants during the dietary evaluation and to help in estimating the portion size of the foods consumed [23].

#### Physical evaluation

Height will be measured without socks and shoes by using a calibrated vertical Seca Portable 217 Stadiometer, to the nearest millimetre. Weight will be measured with light clothing using a Seca 813 digital electronic scale, to the nearest decimal fraction of kilogram. Body mass index (BMI) will be calculated as weight in kilograms divided by the square of height in meters. Body fat composition will be measured using a Tanita portable Body Composition Analyzer SC-240 MA. Waist circumference (WC) and hip circumference (HC) will be measured with a non-elastic Seca measuring tape, to the nearest millimetre. Position of waist circumference measurement will be done at a level midway between the lower rib margin and highest point of iliac crest with the tape all around the body in horizontal position [24]. The hip circumference will be measured at the widest point over the buttock yielding the maximum circumference of the buttocks. For women, this is usually at groin level and for men, it is normally about 2-4 inches below the naval/umbilicus.

The "six minute walk test" will be conducted to measure cardiovascular endurance. The six minute walk test is a sub maximal measure of aerobic capacity [25-27]. A calibrated Jamar hand dynamometer will be used to perform the hand grip strength test. Participants will be asked which the dominant hand is. The first test will be performed with dominant hand and then, with non-dominant hand. Three sets of test will be repeated alternatively for both hands [28,29]. The strength of the hand grip will be recorded as kilogram of force.

### Follow up and outcome measurement

The follow up will be at six months and twelve months for both intervention and control groups. The GPAQ questionnaire survey, 7 days diet history, blood pressure measurement and physical evaluation will be conducted at baseline, six months and twelve months. The log book kept by participants in the intervention group will be checked for completeness and errors after one month of the intervention and the record will be taken at three, six, nine and twelve months in order to check the compliance of self-blood pressure monitoring and home-based exercise, and whether they measured their blood pressured once a week or not.

The primary outcomes will be systolic and diastolic blood pressure measured at zero month (enrolment), six months (end of intervention period), and twelve months (maintenance period). Secondary outcome measures include some Social Cognitive Theory variables related to self-efficacy and self-regulatory such as practice of self-blood pressure monitoring, and adherence of exercise. Other secondary outcomes will be dietary intake, level of physical activity and physical fitness in term of both cardio vascular endurance and muscle strength.

### Data management and analysis

The data analysis will be done by using Stata software (version 11: StataCorp). Bivariate analysis such as Chi-square tests (for dichotomous and categorical variables), t test and ANOVA (for continuous and normally distributed variables) and Mann-Whitney-U test (for continuous variable with skewed distribution) will be applied to compare the two groups with respect of socio-demographic characteristics, number of clinic visits, practice of self-blood pressure monitoring, blood pressure at the time of enrolment, dietary intake, level of physical activity and fitness.

The primary outcome, change in systolic and diastolic blood pressure at six months and twelve months will be compared between and within the groups by using t test and ANOVA. If there are some differences in group characteristics, it will be adjusted by performing multivariate analysis of variance (MANOVA).

Since the secondary outcomes such as numbers of self-blood pressure monitoring, dietary intake, body composition (BMI, fat percentage, waist and hip circumference ratio), level of physical activity and physical fitness are also continuous variables, these will be examined as per primary outcome. The normality of the continuous variables will be checked before analysis and proper transformation will be done if the data is not normally distributed. The level of statistical significance will be set at p < 0.05.

In order to calculate nutrient intake, Nutritionist Pro™ Diet Analysis software will be used to analyse the diet record. It will focus mainly on energy, protein, fat, carbohydrate, sodium, dietary fibre and others. Based on the 7 days diet histories, the average energy consumed with macronutrients (e.g: Energy intake (kcal), protein (g), fat (g), saturated fat (g) and others) and micronutrients (e.g: calcium (mg), iron (mg), vitamins and minerals) value will be calculated. All nutrient intakes will be transferred to the Stata software for final data analysis.

## Ethical consideration

Ethical approval was obtained from the Medical Ethics Committee, University Malaya Medical Centre (Reference Number: 944.18). The trial was registered at Iranian Clinical Trial registry (Reference Number: IRCT2013030512705N1). The control groups will be receiving all components of intervention after completion of the study.

## Discussion

Hypertension is a major contributor to the growing global pandemic of cardiovascular diseases and stroke. It is also one of the main non communicable diseases in Malaysia that are becoming an economic burden of the nation [30]. By realising the health need of the nation, the Malaysian Ministry of Health adopted the National Strategic Plan for Non-Communicable Disease (NSPNCD) in 2010 [31]. The NSPNCD aimed to achieve health promotion and prevention of NCD by increasing awareness of risk factors and adopting a healthy lifestyle in community based approach.

Our intervention is in line with national strategy and approach for prevention and control of hypertension. Previous studies showed that adherence to the "Dietary Approaches to Stop Hypertension (DASH) diet" alone can reduce both systolic and diastolic blood pressure [7,18]. Our lifestyle modification incorporates peer support self-blood pressure monitoring and training for home-based exercise to enhance the effect on blood pressure control. Several previous studies conducted in high income countries used either fixed or mobile phone as a method of delivering health coaching either via phone call or short message [10,32-34]. We selected face to face health coaching and exercise training as an appropriate approach since the education status and functional health literacy is low in our study population. Providing an exercise CD and BMI calculator would also motivate and assist our participants to engage in home-based exercise.

We selected a low income urban community as population of interest in order to reduce existing disparities in health status and access to health care across population sub-groups. Previous research conducted in the study area showed that prevalence of hypertension and other cardiovascular risk factors are higher compared to the nation-wide study. The health system in Malaysia is heavily financed by the Government and all citizens can enjoy financial and geographical access to health care [35]. However, there were several undiagnosed cases of hypertension due to lack of awareness of disease symptoms and irregular medical check-up [3]. In addition, poor treatment adherence and non-compliance behaviour challenged effective hypertension control since many people were unaware of the serious complications of hypertension. A previous study conducted in Malaysia showed that more than 95% of patients were unaware that hypertension requires long term treatment [36].

One of the expert recommendations to improve hypertension management in Asia is to increase awareness of hypertension, enhance treatment adherence and promote home blood pressure monitoring [4]. Positive impact of self-monitoring of blood pressure on treatment adherence and reduction of blood pressure has been demonstrated in high income countries [11,13,37,38].

However, to obtain a blood pressure monitor is a big challenge since more than half of our study population have an average household income less than 2,000 MYR which equivalent to (USD 605). The purchase of a reliable quality digital blood pressure monitor would cost about 10% of household income which is generally considered as catastrophic health care expenditure [39]. Our study proposed a solution to overcome the issue of unaffordability to obtain the blood pressure monitor. Since there is an existing social network in the community and practice of sharing resources, we formed a peer support group and provided a digital blood pressure monitor. We believed that it would encourage the sharing of knowledge on hypertension, disease progress and practice of self-blood pressure monitoring among the participants. Although it is expensive for a single person to purchase a blood pressure monitor for his/her own, sharing equipment among peer group members would be affordable and a practical solution for a low income community who lives the same community housing complex.

We expected that after completing intervention, the participants of our study could benefit by increasing awareness of healthy diet and physical activity, adopting healthy life style and improving self-management of hypertension. The proposed intervention would ensure the self-efficacy and self-regulation of regular blood pressure monitoring, healthy diet choice, and maintaining physical activities and exercise with peer social support. There would be a promising possibility that our intervention is widely replicable and adopted as a model of community based approach for combating hypertension in low and middle income countries.

## Competing interests

The authors declare that they have no competing interests.

## Authors' contributions

TTS, HAM and AMN conceived the study and designed the interventions. AB and LJM gave input on method and statistical analysis. NAA, FMH, NT and MD conducted the pilot testing of the study tools. All authors were responsible for the drafting of this paper and approved the final manuscript.
