# Correction to: The effectiveness of a life style modification and peer support home blood pressure monitoring in control of hypertension: protocol for a cluster randomized controlled trial

**DOI:** 10.1186/s12889-017-4862-y

**Published:** 2017-11-06

**Authors:** Tin Tin Su, Hazreen Abdul Majid, Azmi Mohamed Nahar, Nurul Ain Azizan, Farizah Mohd Hairi, Nithiah Thangiah, Maznah Dahlui, Awang Bulgiba, Liam J. Murray

**Affiliations:** 1 Centre for Population Health (CePH), Department of Social and Preventive Medicine, Faculty of Medicine, University of Malaya, Kuala Lumpur, Malaysia; 2 Department of Sports Medicine, Faculty of Medicine, University of Malaya, Kuala Lumpur, Malaysia; 3 Julius Centre University of Malaya (JCUM), Department of Social and Preventive Medicine, Faculty of Medicine, University of Malaya, Kuala Lumpur, Malaysia; 4 Centre for Public Health, Queen’s University of Belfast, Belfast, Ireland

## Correction

After publication of the article [1], it has been brought to our attention that the methodology outlined in the original article was not able to be fully carried out. The article planned a two armed randomized control trial. However, due to a lower response than expected and one housing complex dropping out from the study, the method was changed to pre- and post-intervention with no control group. All other methods were conducted as outlined in the original article.
